# Supplementary material for: What Can We Learn from FGF-2 Isoform-Specific Mouse Mutants? Differential Insights into FGF-2 Physiology In Vivo
Source: Int J Mol Sci. 2020 Dec 31;22(1):390. doi: 10.3390/ijms22010390 (PMC7795026; doi:10.3390/ijms22010390)
Supplement: Supplementary file 1 [file ijms-22-00390-s001.pdf]

Strain	**FGF-2LMWko**<br>(FGF-2tm2Doe)	**FGF-2HMWko**<br>(FGF-2tm3Doe)	**FGF-2HMWtg**<br>Overexpressed human 24 kDa driven by PGK promoter	**FGF-2LMWtg**<br>Overexpressed rat 18 kDa driven by RSV promoter
**Ischemia-reperfusion injury models**	♂♀ (*ex vivo*)<br>↑ phosphorylated JNK, MKK4, MKK7 and c-Jun protein level<br>↓ recovery of contractile heart function partly improved by inhibition of the JNK pathway<br>↑ apoptosis (caspase 3 and TUNEL⁺ cells) after DMSO treatment<br>(Liao, Porter et al. 2007)[1]	♂♀<br>↑ recovery of contractile and diastolic function<br>(Liao, Bodmer et al. 2010)[2]<br>♂♀<br>↑ postischemic recovery of contractile function<br>↑ levels of phosphorylated PKCα at ischemia onset<br>↑ levels of myofilament PKCα in ischemia<br>↓ levels of myofilament PKCδ in ischemia<br>↑ phosphorylation of troponin I and T in ischemia and early reperfusion<br>↑ levels of phosphorylated PKCε at early reperfusion<br>↑ activity of actomyosin MgATPase in reperfusion<br>↑ levels of myofilament PKCε after reperfusion<br>↑ levels of myofilament PKCδ after reperfusion<br>↓ recovery by PKCε inhibition<br>↓ PKCα dependent myofilament sensitivity against calcium<br>**FGF-2^{tm3Doe} x PKCαko**<br>↓ cardioprotection<br>↑ systolic and diastolic dysfunction<br>↑ amount of PKCε expression<br>(Manning, Perkins et al. 2013)[3]	♂♀ (*ex vivo*)<br>↓ recovery of contractile and relaxation function<br>(Liao, Bodmer et al. 2010)[2]	♂♀ (*ex vivo*)<br>↑ myocyte viability (↓ LDH activity) after reperfusion<br>(Sheikh, Sontag et al., 2000)[4]
**Isoproterenol model of cardiac hypertrophy**	♂ **following treatment**<br>↑ fibrosis<br>↑ Col1 and α-SMA protein level<br>♀ **following treatment**<br>↑ but attenuated cardiac hypoplasia (↓ H/B ratio)<br>(Nusayr, Sadideen et al. 2013)[5]	♂ **following treatment**<br>↑ but attenuated cardiac hypertrophy (↑ H/B ratio)<br>↓ fibrosis<br>↑ α-SMA and ANF expression<br>♀ **following treatment**<br>↓ cardiac hypertrophy<br>(Nusayr, Sadideen et al. 2013)[5]		
**Doxorubicin model of acute cardiac injury**		♂♀ **following treatment**<br>Sex independent cardioprotection (no changes in any echocardiographic parameters or Bnip3 protein level)<br>(Koleini, Santiago et al. 2019)[6]		

Supplement Table S1. Evaluation of male (♂) and female (♀) FGF-2 isoform-specific mouse mutants in ischemic-reperfusion injury, isoproterenol model of cardiac hypertrophy and doxorubicin model of acute cardiac injury. Results were displayed as increased (↑) or decreased (↓) for either FGF-2 isoform-specific ko mice (FGF-2LMWko and FGF-2HMWko), or mice additionally overexpressing rat FGF-2LMW (FGF-2LMWtg) or human 24 kDa FGF-2HMW (FGF-2HMWtg) compared to wildtype littermates.

α-SMA, α-smooth-muscle actin; ANF, atrial natriuretic factor; Col1a1, type 1 collagen; DMSO, dimethyl sulfoxid; FGF-2, fibroblast growth factor 2; JNK, c-Jun N-terminal kinase; ko, knock out; H/B, heart to body weight ratio; HMW, high molecular weight; LDH, lactate dehydrogenase; LWW, low molecular weight; MAPK, mitogen activated protein kinase; MKK, Mitogen-activated protein kinase kinase; PKC, protein kinase C; TUNEL, TdT-mediated dUTP-biotin nick end labeling; +, positive.

Strain	**FGF-2LMWko**<br>(FGF-2tm2Doe)	**FGF-2HMWko**<br>(FGF-2tm3Doe)	**FGF-2HMWtg**<br>Overexpressed human 22, 23, 24 kDa driven by Col3.6 promoter	**FGF-2LMWtg**<br>Overexpressed human 18 kDa driven by Col3.6 promoter
**Phenotype**	♂<br>↓ vertebral bone mineral density and content<br>↑ sFRP1 protein levels in trabecular bones<br>(Xiao, Liu et al. 2009)[7]	♂<br>↑ whole body bone mineral density and content<br>↑ vertebral, femoral bone mineral density and content<br>↑ femoral bone volume, trabecular thickness, number (cortical bone area, thickness, cortical mask)<br>↓ femoral trabecular spacing<br>↑ connective tissue density<br>↓ cortical porosity, bone resorption (↓ osteoclast surface, number)<br>↑ bone formation in cortical periosteum, trabecular bone (↑ osteoblast surface, inter-label thickness, mineral apposition rate)<br>↑ tibial *Col1a1*, *Runx2*, *osterix*, *oc*, *op*, *Dmp1* gene expression<br>↓ femoral *Sost* gene expression<br>↓ serum sclerostin, protein levels<br>↓ tibial *Fgf-2*, *Fgf-23* gene expression<br>(Homer-Bouthiette, Doetschman et al. 2014)[8]	♂<br>dwarfism, osteomalacia<br>↓ body weight<br>↓ whole body bone mineral density and content<br>↓ femoral bone length<br>↓ vertebral volume, bone mineral density and content<br>↓ femoral bone volume, trabecular number, thickness<br>↑ femoral trabecular spacing<br>↑ bone resorption (↑ osteoclast surface, number)<br>↓ bone formation (↓ osteoblast, mineralization surface, bone formation rate)<br>↓ tibial *Col1a1*, *Oc* gene expression<br>↑ tibial *Op*, *Mgp* gene expression<br>↓ serum phosphate<br>↑ serum PTH, CTX, FGF-23<br>↑ tibial, femoral *Fgf-23*, *Phex* gene expression, protein levels<br>↑ renal *Fgfr-1c*, *Fgfr-3c*, *Klotho* gene expression<br><br>**♂ with continuous phosphate diet**<br>↑ body weight, bone mineral content, bone mineral density<br>↑ serum phosphate to a normal level<br>↑ serum FGF-23<br>(Xiao, Naganawa et al. 2010)[9]<br>♂<br>↓ whole body bone mineral density and content<br>↓ femoral, tibia, vertebral bone mineral density and content<br>↓ serum phopshate<br>↑ serum FGF-23, PTH<br>↑ renal *Fgfr-3c* gene expression<br>↑ renal FGFR-1, FGFR-3, Klotho, C-Fos, activated ERK protein levels<br>↑ renal *Klotho, cFos, egr1* gene expression<br>↓ renal *Npt2* gene expression<br>↑ renal *Cyp24, Cyp27b1* gene expression<br>↓ renal Npt2 protein levels<br>(Du, Xiao et al. 2017)[10]	♂<br>↑ vertebral, tibial, femoral bone mineral density and content<br>↑ femoral bone volume, trabecular thickness, cortical bone area, thickness<br>↓ *sFRP1* gene expression, protein levels in trabecular bones<br>↑ *β-catenin* gene expression, protein levels<br>(Xiao, Liu et al. 2009)[7]<br>♂<br>↑ *Fgfr-1*, *Fgfr-2*, *oc*, *β-catenin* gene expression in calvaria bone<br>↓ *sFRP1* gene expression in calvaria bone<br>↑ calvarial inter-label thickness, mineral apposition rate<br>(Xiao, Ueno et al. 2014)[11]

**Strain**	**FGF-2LMWko**<br>(FGF-2^{tm2Doe})	**FGF-2HMWko**<br>(FGF-2^{tm3Doe})	**FGF-2HMWtg**<br>Overexpressed human 22, 23, 24 kDa driven by Col3.6 promoter	**FGF-2LMWtg**<br>Overexpressed human 18 kDa driven by Col3.6 promoter
**Phenotype**			♂<br>dwarfism<br>↓ body weight, tail length<br>↓ whole body bone mineral density and content<br>↓ femoral bone mineral density, length<br>↓ vertebral bone mineral density<br>↓ serum phosphate<br>↑ serum FGF-23, PTH<br>↑ urinary phosphate level<br>↓ renal *Npt2* gene expression<br>↑ renal Klotho, activated renal ERK protein levels<br>↑ cortical porosity, trabecular spacing, osteoid volume<br>↓ cortical thickness, tissue<br>↓ endosteal/periosteal perimeter, subendosteal area<br>↓ mineralization of cortical bone area, metaphyseal cancellous bone volume, trabecular number<br>↑ osteoclast number, surface<br>↑ femoral *Fgfr-3c*, *Pthr1*, *Op*, *Fgf23*, *Mgp* gene expression<br>(Xiao, Du et al. 2017)[12]<br>♀<br>↓ body weight<br>↓ femoral, tibial, vertebral bone mineral density and content<br>↓ serum phosphate<br>↑ serum FGF-23, 1,25D<br>↑ urinary phosphate level<br>↑ renal FGFR-1, En-1, klotho protein levels<br>↑ renal *Klotho*, *Sostdc-1*, *En-1*, *Cyp24* gene expression<br>↑ activated renal ERK, Gsk-3β (Tr216) protein levels<br>↓ renal *Npt2*, *Akt* gene expression<br>↓ activated renal Gsk-3β (Ser9), active β-catenin and Akt protein levels<br>(Du, Xiao et al. 2016)[13]	

Strain	FGF-2LMWko<br>(FGF-2tm2Doe)	FGF-2HMWko<br>(FGF-2tm3Doe)	FGF-2HMWtg<br>Overexpressed human 22, 23, 24 kDa driven by Col3.6 promoter	FGF-2LMWtg<br>Overexpressed human 18 kDa driven by Col3.6 promoter
			♀<br>↓ body weight<br>↓ femoral, vertebral bone mineral density and content<br>↓ femur length, cortical density, mineral apposition rate<br>↑ cortical porosity<br>↓ femoral bone volume, trabecular number<br>↑ femoral trabecular spacing<br>↑ osteoid volume<br>↑ serum FGF-23, ALP<br>↓ serum phosphate, TNAP<br>↓ TNAP activity in osteocytes<br>↑ renal *Fgfr-1c*, *Fgfr-3* gene expression<br>↓ renal *Npt2a* gene expression<br>↑ tibia *Fgf-2*, *Fgfr-1c*, *Col1a1*, *Mgp*, *Dmp4*, *Phex*, *Mepe*, *Enpp1*, *SLc20a1* gene expression<br>↓ tibia *Dmp1*, *Rankl*, *Oc* gene expression<br>↑ femur cortical ERK, FGFR-1 protein levels<br>(Xiao, Homer-Bouthiette et al. 2018)[14]	

Supplement table S2A. Extensive characterization of the bone related phenotype of adult FGF-2 isoform-specific mouse mutants in chronological order. All data of either FGF-2LMWko, FGF-2HMWko or mice additionally overexpressing human FGF-2LMW (FGF-2LMWtg) or FGF-2HMW (FGF-2HMWtg) were listed as increased (↑) or decreased (↓) compared to wt littermates. Whenever possible results were separated for male (♂) and female (♀) mice.

1,25D, 1,25-dihydroxyvitamin D; ALP, alkaline phosphatase; Col1a1, Type I collagen; CTX, c-terminal telopeptide of type 1 collagen; Cyp24, renal 25-hydroxyvitamin D 24-hydroxylase; Cyp27b1, renal 25-hydroxyvitamin D 1alpha-hydroxylase; Dmp, Dentin matrix phosphoprotein; Egr-1, early growth response-1 transcription factor; En-1, Engrailed-1; Enpp1, Ectonucleotide pyrophosphatase/phosphodiesterase family member 1; FGF, fibroblast growth factor; FGFR, fibroblast growth factor receptor; Gsk3β, Glycogen Synthase Kinase 3 Beta; HMW, high molecular weight; knock out; LWW, low molecular weight; Mepe, Matrix extracellular phosphoglycoprotein; Mgp, Matrix gla protein; Npt2, sodium phosphate co transporter; Oc, Osteocalcin; Op, Osteopontin; Phex, Phosphate-regulating neutral endopeptidase; PTH, Parathyroid hormone; PTHR1, parathyroid hormone 1 receptor; Runx2, runt-related transcription factor 2; sFRP-1, secreted frizzled receptor 1; Slc20a1, Sodium-dependent phosphate transporter 1; Sost, sclerostin; Sostdc-1, Sclerostin domain-containing-1; tg, transgene; TNAP, tissue nonspecific alkaline phosphatase.

Strain	**FGF-2LMWko**<br>(FGF-2^{tm2Doe})	**FGF-2HMWko**<br>(FGF-2^{tm3Doe})	**FGF-2HMWtg**<br>Overexpressed human 22, 23, 24 kDa driven by Col3.6 promoter	**FGF-2LMWtg**<br>Overexpressed human 18 kDa driven by Col3.6 promoter
**Aging/Osteoarthritis**	♂♀<br>↑ OA in knee joints (flattening of tibial plateau, osteophyte formation)<br>♂<br>↓ femoral, tibial bone volume, trabecular number, thickness<br>↑ femoral, tibial trabecular spacing<br>↓ proteoglycan content, cartilage thickness in knee joint<br>↑ tendonitis, arthritis<br>↑ MMP-13, ADAMTS-5, FGF-2, FGF-23, FGFR-1 protein levels in articular cartilages<br>↑ *Igf1, IL-1β, Bmp4, Hif1α, Bax, Fgf-2, Fgf-23, Fgfr-3 Vegf, Col10* gene expression in knee joints<br>↑ activated ERK protein levels in articular cartilage<br>↓ activated FGFR-3 in articular cartilage<br>↑ signs of OA following tibial loading (loss of proteoglycan content, thinning of subchondral bone)<br>(Burt, Xiao et al. 2019)[15]	♂♀<br>no radiographical signs of OA in knee joints<br>♂<br>↑ activated FGFR-3 protein levels in knees<br>↓ FGF-2 protein levels in articular cartilage<br>(Burt, Xiao et al. 2019)[15]	♂<br>↑ OA in knee joints (flattening of tibial plateau, osteophyte formation, femoral subchondral bone thinning, sclerotic bone development, narrowing of the patellofemoral space, loss of trabeculae, sclerosis of femur)<br>↓ epiphyseal bone volume density, trabecular thickness, number in femur, tibiae<br>↓ proteoglycan content, cartilage thickness in knee joint<br>↑ *MMP13*, *Col10*, *ADAMTS-5* gene expression in articular cartilages<br>↑ *Igf1, IL-1β, Bmp2, Bmp4, Hif1α, Bax, Sox9, Vegf* gene expression in knee joints<br>↑ FGF-23, FGFR-1 protein levels in knee joints<br>↓ mineralization of hypertrophic chondrocytes<br>(Meo Burt, Xiao et al. 2016)[16]<br>♂<br>↓ *Sost*, *Dkk1*, *Lrp6* gene expression in knee joints<br>↑*Wnt5a*, *Axin2*, *Lef1* gene expression in knee joints<br>↓ Sost, Lrp6 protein levels in knee joints<br>↑ Wnt7b, Wnt5a, Lrp5, Axin2, Gsk-3β, Lef1, nuclear β-catenin protein levels in knee joints<br>↑ *Col10*, *Mmp9*, *Mmp13* gene expression in femoral cartilage<br>(Meo Burt, Xiao et al. 2018)[17]<br>♂♀<br>↑ signs of OA in knee joints (flattening of tibial plateau, osteophyte formation, sclerosis)<br>↓ femoral, tibial bone volume, trabecular number, thickness<br>↓ proteoglycane content, cartilage thickness in knee joints<br>↑ cartilage calcification in knee cartilage<br>♂<br>↑ *Fgfr-1c*, *Fgf-18*, *Col10*, *Mmp13* gene expression in knee joints<br>↓ *Fgfr-3c* gene expression in knee joints<br>↑ FGF-2, FGF-23, FGFR-1 protein level in subchondral bone<br>↑ MMP13, SOX9, ADAMTS-5 protein level in articular cartilages<br>↓ Dkk1 Lrp6, Sost protein levels in articular cartilage<br>↑ Wnt7b, Lrp5, Gsk-3β, active β-catenin, AXIN2 protein levels in articular cartilage<br>(Xiao, Williams et al. 2020)[18]	♂<br>no radiographical signs of OA in knee joints<br>(Meo Burt, Xiao et al. 2016)[16]

Supplement table S2B. Extensive characterization of the bone related phenotype developed through aging of FGF-2 isoform-specific male (♂) and female (♀) mouse mutants. All alterations through aging were listed for FGF-2LMWko, FGF-2HMWko, FGF-2LMWtg or FGF-2HMWtg mouse mutants given as increased (↑) or decreased (↓) compared to wt littermates

Adamts5, A disintegrin metalloproteinase with thrombospondin motifs 5; Bax, B-cell lymphoma 2 associated X, apoptosis regulator; BMP, Bone morphogenetic protein ; Col10, type 10 collagen; Dkk1, Dickkopf-Like Protein 1; FGF, fibroblast growth factor; FGFR, fibroblast growth factor receptor; Gsk3β, Glycogen Synthase Kinase 3 Beta; Hif1α, hypoxia inducible factor 1; HMW, high molecular weight; IL-1β, interleukin-1 β; Igf1, insulin like growth factor 1; ko, knock out; Lef1, Lymphoid Enhancer Binding Factor 1; LWW, low molecular weight; Lrp, low density lipoprotein receptor-related protein; Mmp, Matrix metallopeptidase; OA, Osteoarthritis; Sox9, Sex-determining region Y box 9; Sost, sclerostin; tg, transgene; Vegf, vascular endothelial growth factor; Wnt, wingless-type.

Strain	**FGF-2LMWtg**<br>Overexpressed human 18 kDa driven by Col3.6 promoter
Fracture healing	♂ **calvarial defect model**<br>↑ healing of calvaria defect<br>↑ bone volume, formation<br>↑ healing after BMP-2 addition<br><br>**additional BMP-2 treatment:**<br>↑ bone volume, mineral apposition rate<br>↑ healing of calvaria defects<br>↑ *Fgf-2*, *Bmp-2*, *Fgfr-1*, *Fgfr-2*, *Runx2*, *osterix*, *Oc*, *Lrp5*, *Wnt10b*, *β-catenin* gene expression in calvaria bone<br>(Xiao, Ueno et al. 2014)[11]<br><br>♀ **closed tibial fracture model**<br>↑ tibial fracture healing<br>↓ fracture callus expansion<br>↑ fracture callus remodeling process<br>↑ *Fgf-2* gene expression in callus osteoblasts, osteocytes<br>↓ *Fgf-18* gene expression<br>↑ *Fgfr-1, Fgfr-3* mRNA earlier through fracture healing process<br>↑ *Sox9* gene, protein expression in fracture callus<br>↓ C*ol2a1* gene, protein expression in fracture callus<br>↑ *Pdgfb, Pdgfd* gene, protein expression in fracture callus, earlier peak of *Pdgfc* gene, protein expression through healing<br>↑ *Col10, Mmp9* gene, protein expression earlier through fracture healing process<br>↑ *Vegf* mRNA, protein in bone marrow<br>↑ *Trap, cathepsin* gene, protein expression peaked earlier in osteoclasts, chondroclasts<br>↑ *Runx2, osterix, Oc* gene, protein expression peaked earlier in periosteum, osteoblasts<br>(Hurley, Adams et al. 2016)[19]

Supplement table S2C. Evaluation of FGF-2LMWtg mice different models of fracture healing. Male (♂) and female (♀) mice were analyzed and results were displayed as increased (↑) or decreased (↓) compared to wt littermates.

BMP, Bone morphogenetic protein; Col, collagen; FGF, fibroblast growth factor; FGFR, fibroblast growth factor receptor; LWW, low molecular weight; Lrp, low density lipoprotein receptor-related protein; Mmp, Matrix metallopeptidase; Oc, Osteocalcin; Pdgf, Platelet-derived growth factor; Runx2, runt-related transcription factor 2; Sox9, Sex-determining region Y box 9; Trap, tartrate resistant acid phosphatase; Vegf, vascular endothelial growth factor; Wnt, wingless-type.

**Strain**	**FGF-2HMWtg**<br>Overexpressed human 22, 23, 24 kDa driven by Col3.6 promoter	
**FGF-23 antibody treatment**	**Short-term treatment**	**Long-term treatment**
	♂<br>↓ renal *Fgfr-3*, *klotho*, *egr1*, *c-fos* gene expression<br>↓ renal FGFR-1, FGFR-3 protein levels<br>↑ renal *Npt2a, Cyp24* gene expression<br>↑ serum phosphate, 1,25D<br>↓ urinary phosphate<br>(Du, Xiao et al. 2017)[10]<br><br>♀<br>↓ renal *Fgfr-1c*, *Fgfr-3c, Cyp24* gene expression<br>↑ renal *Npt2a, Cyp27b1*, gene expression<br>↑ serum phosphate, 1,25D<br>↓ urinary phosphate<br>(Xiao, Homer-Bouthiette et al. 2018)[14]	♀<br>↑ vertebral bone mineral content, density, femoral bone mineral content<br>↑ femoral bone length<br>↓ femoral cortical porosity, trabecular spacing<br>↑ femoral bone volume density, trabecular number, intralabel thickness<br>↑ femoral mineral apposition rate<br>↓ femoral mineralization defects<br>↓ femur cortical ERK, FGFR-1 protein levels<br>↓ tibia *Fgf-2* gene expression<br>↑ tibia *Fgfr-1c, Dmp4, Opg, Runx2, Col1a1, Oc, Opn, Phex, Mepe, Enpp1, Ank, Slc20a1* gene expression<br>↑ serum phosphate<br>↓ serum ALP<br>↑ serum TNAP<br>↓ urinary phosphate<br>(Xiao, Homer-Bouthiette et al. 2018)[14]<br><br>♀<br>↓ signs of OA in knee joints (flattening of tibial plateau, thinning of femoral subchondral bone, cartilage thickness)<br>↑ femoral bone volume, trabecular number, thickness<br>↓ femoral trabecular spacing<br>↑ articular cartilage thickness<br>↓ *Col10*, *Mmp9*, *Mmp13* gene expression in femoral cartilage<br>↓ MMP9, MMP13 protein levels in articular cartilage<br>(Meo Burt, Xiao et al. 2018)[17]

Supplement table ~~S2C~~S2D. Effects following FGF-23 antibody treatment in male (♂) and female (♀) FGF-2HMWtg mice. All measurements were conducted 24 hours following single injection (short-term treatment) of a FGF23 neutralizing antibody (10 mg/kg) or after repeated treatments with the same dosage over six weeks (long-term treatment). Results were shown as increased (↑) or decreased (↓) compared to vehicle treated littermates.

1,25D, 1,25-dihydroxyvitamin D; ALP, alkaline phosphatase; Col10, Type 10 collagen; Cyp24, renal 25-hydroxyvitamin D 24-hydroxylase; Cyp27b1, renal 25-hydroxyvitamin D 1alpha-hydroxylase; Dmp, Dentin matrix phosphoprotein; Egr-1, early growth response-1 transcription factor; En-1, Engrailed-1; Enpp1, Ectonucleotide pyrophosphatase/phosphodiesterase family member 1; FGF, fibroblast growth factor; FGFR, fibroblast growth factor receptor; HMW, high molecular weight; knock out; LWW, low molecular weight; Mepe, Matrix extracellular phosphoglycoprotein; Mmp, Matrix metallopeptidase; Npt2, sodium phosphate co transporter; Oc, Osteocalcin; Op, Osteopontin; Phex, Phosphate-regulating neutral endopeptidase; PTH, Parathyroid hormone; Runx2, runt-related transcription factor 2; Slc20a1, Sodium-dependent phosphate transporter 1; tg, transgene; TNAP, tissue nonspecific alkaline phosphatase.

Strain	FGF-2HMWtg<br>Overexpressed human 22, 23, 24 kDa driven by Col3.6 promoter	
	**Short-term treatment**	**Long-term treatment**
**FGFR Inhibitor NVP-BGJ398**	♀<br>↓ renal FGFR-1, Sostdc-1, En-1, klotho protein levels<br>↓ activated renal ERK, β-catenin, Gsk-3b (Tyr216) protein levels<br>↑ renal *Npt2a* gene expression, protein levels<br>↑ renal activated Gsk-3b (Ser 9), active β-catenin, Akt protein levels<br>↑ renal *Cyb24*, *Akt*, *b-catenin*, *Cyp27b1* gene expression<br>↓ renal *Sostdc-1*, *En-1* gene expression<br>↑ serum phosphate, PTH, 1,25D, FGF-23, Klotho<br>↓ urinary phosphate<br>(Du, Xiao et al. 2016)[13]<br><br>♂<br>↑ femoral bone mineral density<br>↓ femoral *Fgf-23* gene expression<br>↑ renal *Npt2* gene expression<br>↑ serum phosphate, αKlotho, 1,25D<br>↓ serum creatinine, FGF-23<br>↓ urinary phosphate<br>(Xiao, Du et al. 2017)[12]	♂<br>↓ dwarfism<br>↓ body weight<br>↑ tail length, femoral trabecular thickness, density<br>↓ femoral, excised vertebrae bone mineral density<br>↓ femoral *Fgfr-3c* gene expression<br>↑ femoral *Fgfr-1c*, *Mepe*, *Op*, *Dmp1*, *Bsp*, *Pthr1* gene expression<br>↓ disorganized growth of femoral plates, trabecular spacing<br>↑ bone formation rate, osteoblast activity<br>↑ femoral osteoclast number, surface<br>↑ integrity of femoral cortical bone<br>↑ endosteal, periosteal perimeter, subendosteal area<br>↓ cortical porosity<br>↑ cortical thickness, tissue<br>↑ activated renal Npt2 protein levels<br>↓ activated renal ERK protein levels<br>↑ serum phosphate, FGF-23<br>↑ urinary phosphate<br>(Xiao, Du et al. 2017)[12]<br><br>♂♀<br>↓ flattened tibia plateau, narrowing of joint space, osteophytes, uneven joint surface<br>↑ cartilage thickness in knee joint<br>↓ cartilage calcification in knee joint<br>↓ irregular shape, thinning of subchondral bone<br><br>♂<br>↑ femoral trabecular thickness, bone volume<br>↓ *Mmp13*, *Sox9*, *ADAMTS-5* gene expression in articular cartilages<br>↓ *Fgf-18*, *Col10*, *Fgfr-3c* gene expression in knees<br>↓ activated FGFR-1, Gsk3b, β-catenin, Lrp5 protein levels in articular cartilage<br>↓ FGF-23, Axin2, Wnt7b protein levels in articulate cartilage<br>↑ Dkk1 protein levels in articulate cartilage<br>↑ Sost protein levels in subchondral bone of knee joints<br>↓ serum FGF-23<br>(Xiao, Williams et al. 2020)[18]

Supplement table ~~S2D~~S2E. Effects following administration of the FGFR inhibitor NVP-BGJ398 in male (♂) and female (♀) FGF-2HMWtg mice. All measurements were conducted 24 hours following single oral administration of the FGFR inhibitor NVP-BGJ398 with 50 mg/kg (short-term treatment) or following daily subcutaneous injection of the same antibody (2 mg/kg) for at least six weeks (long-term treatment). Results were shown as increased (↑) or decreased (↓) compared to vehicle treated littermates.

1,25D, 1,25-dihydroxyvitamin D; ALP, alkaline phosphatase; Bsp, bone sialoprotein; Col10, Type 10 collagen; Cyp24, renal 25-hydroxyvitamin D 24-hydroxylase; Cyp27b1, renal 25-hydroxyvitamin D 1alpha-hydroxylase; Dkk1, Dickkopf-Like Protein 1; Dmp, Dentin matrix phosphoprotein; Egr-1, early growth response-1 transcription factor; En-1, Engrailed-1; Enpp1, Ectonucleotide pyrophosphatase/phosphodiesterase family member 1; FGF, fibroblast growth factor; FGFR, fibroblast growth factor receptor; Gsk3β, Glycogen Synthase Kinase 3 Beta; HMW, high molecular weight; knock out; LWW, low molecular weight; Mepe, Matrix extracellular phosphoglycoprotein; Mmp, Matrix metallopeptidase; Npt2, sodium phosphate co transporter; Oc, Osteocalcin; Op, Osteopontin; Phex, Phosphate-regulating neutral endopeptidase; PTH, Parathyroid hormone; Pthr1, parathyroid hormone 1 receptor; Runx2, runt-related transcription factor 2; Slc20a1, Sodium-dependent phosphate transporter 1; Sost, sclerostin; tg, transgene; TNAP, tissue nonspecific alkaline phosphatase; Wnt, wingless-type.

1. Liao, S.; Porter, D.; Scott, A.; Newman, G.; Doetschman, T.; Schultz Jel, J. The cardioprotective effect of the low molecular weight isoform of fibroblast growth factor-2: the role of JNK signaling. *J. Mol. Cell. Cardiol.* **2007**, *42*, 106-120, doi:10.1016/j.yjmcc.2006.10.005.
2. Liao, S.; Bodmer, J.R.; Azhar, M.; Newman, G.; Coffin, J.D.; Doetschman, T.; Schultz Jel, J. The influence of FGF2 high molecular weight (HMW) isoforms in the development of cardiac ischemia-reperfusion injury. *J. Mol. Cell. Cardiol.* **2010**, *48*, 1245-1254, doi:10.1016/j.yjmcc.2010.01.014.
3. Manning, J.R.; Perkins, S.O.; Sinclair, E.A.; Gao, X.; Zhang, Y.; Newman, G.; Pyle, W.G.; Schultz Jel, J. Low molecular weight fibroblast growth factor-2 signals via protein kinase C and myofibrillar proteins to protect against postischemic cardiac dysfunction. *Am J Physiol Heart Circ Physiol* **2013**, *304*, H1382-1396, doi:10.1152/ajpheart.00613.2012.
4. Sheikh, F.; Sontag, D.P.; Fandrich, R.R.; Kardami, E.; Cattini, P.A. Overexpression of FGF-2 increases cardiac myocyte viability after injury in isolated mouse hearts. *Am J Physiol Heart Circ Physiol* **2001**, *280*, H1039-1050, doi:10.1152/ajpheart.2001.280.3.H1039.
5. Nusayr, E.; Sadideen, D.T.; Doetschman, T. FGF2 modulates cardiac remodeling in an isoform- and sex-specific manner. *Physiol Rep* **2013**, *1*, doi:10.1002/phy2.88.
6. Koleini, N.; Santiago, J.J.; Nickel, B.E.; Sequiera, G.L.; Wang, J.; Fandrich, R.R.; Jassal, D.S.; Dhingra, S.; Kirshenbaum, L.A.; Cattini, P.A., et al. Elimination or neutralization of endogenous high-molecular-weight FGF2 mitigates doxorubicin-induced cardiotoxicity. *Am J Physiol Heart Circ Physiol* **2019**, *316*, H279-H288, doi:10.1152/ajpheart.00587.2018.
7. Xiao, L.; Liu, P.; Li, X.; Doetschman, T.; Coffin, J.D.; Drissi, H.; Hurley, M.M. Exported 18-kDa isoform of fibroblast growth factor-2 is a critical determinant of bone mass in mice. *J Biol Chem* **2009**, *284*, 3170-3182, doi:10.1074/jbc.M804900200.
8. Homer-Bouthiette, C.; Doetschman, T.; Xiao, L.; Hurley, M.M. Knockout of nuclear high molecular weight FGF2 isoforms in mice modulates bone and phosphate homeostasis. *J Biol Chem* **2014**, *289*, 36303-36314, doi:10.1074/jbc.M114.619569.
9. Xiao, L.; Naganawa, T.; Lorenzo, J.; Carpenter, T.O.; Coffin, J.D.; Hurley, M.M. Nuclear isoforms of fibroblast growth factor 2 are novel inducers of hypophosphatemia via modulation of FGF23 and KLOTHO. *J Biol Chem* **2010**, *285*, 2834-2846, doi:10.1074/jbc.M109.030577.
10. Du, E.; Xiao, L.; Hurley, M.M. FGF23 Neutralizing Antibody Ameliorates Hypophosphatemia and Impaired FGF Receptor Signaling in Kidneys of HMWFGF2 Transgenic Mice. *J Cell Physiol* **2017**, *232*, 610-616, doi:10.1002/jcp.25458.
11. Xiao, L.; Ueno, D.; Catros, S.; Homer-Bouthiette, C.; Charles, L.; Kuhn, L.; Hurley, M.M. Fibroblast growth factor-2 isoform (low molecular weight/18 kDa) overexpression in preosteoblast cells promotes bone regeneration in critical size calvarial defects in male mice. *Endocrinology* **2014**, *155*, 965-974, doi:10.1210/en.2013-1919.
12. Xiao, L.; Du, E.; Homer-Bouthiette, C.; Hurley, M.M. Inhibition of FGFR Signaling Partially Rescues Hypophosphatemic Rickets in HMWFGF2 Tg Male Mice. *Endocrinology* **2017**, *158*, 3629-3646, doi:10.1210/en.2016-1617.
13. Du, E.; Xiao, L.; Hurley, M.M. FGFR Inhibitor Ameliorates Hypophosphatemia and Impaired Engrailed-1/Wnt Signaling in FGF2 High Molecular Weight Isoform Transgenic Mice. *J Cell Biochem* **2016**, *117*, 1991-2000, doi:10.1002/jcb.25493.
14. Xiao, L.; Homer-Bouthiette, C.; Hurley, M.M. FGF23 Neutralizing Antibody Partially Improves Bone Mineralization Defect of HMWFGF2 Isoforms in Transgenic Female Mice. *J. Bone Miner. Res.* **2018**, *33*, 1347-1361, doi:10.1002/jbmr.3417.
15. Burt, P.M.; Xiao, L.; Doetschman, T.; Hurley, M.M. Ablation of low-molecular-weight FGF2 isoform accelerates murine osteoarthritis while loss of high-molecular-weight FGF2 isoforms offers protection. *J Cell Physiol* **2019**, *234*, 4418-4431, doi:10.1002/jcp.27230.
16. Meo Burt, P.; Xiao, L.; Dealy, C.; Fisher, M.C.; Hurley, M.M. FGF2 High Molecular Weight Isoforms Contribute to Osteoarthropathy in Male Mice. *Endocrinology* **2016**, *157*, 4602-4614, doi:10.1210/en.2016-1548.
17. Meo Burt, P.; Xiao, L.; Hurley, M.M. FGF23 Regulates Wnt/beta-Catenin Signaling-Mediated Osteoarthritis in Mice Overexpressing High-Molecular-Weight FGF2. *Endocrinology* **2018**, *159*, 2386-2396, doi:10.1210/en.2018-00184.
18. Xiao, L.; Williams, D.; Hurley, M.M. Inhibition of FGFR Signaling Partially Rescues Osteoarthritis in Mice Overexpressing High Molecular Weight FGF2 Isoforms. *Endocrinology* **2020**, *161*, doi:10.1210/endocr/bqz016.
19. Hurley, M.M.; Adams, D.J.; Wang, L.; Jiang, X.; Burt, P.M.; Du, E.; Xiao, L. Accelerated fracture healing in transgenic mice overexpressing an anabolic isoform of fibroblast growth factor 2. *J Cell Biochem* **2016**, *117*, 599-611, doi:10.1002/jcb.25308.
